# Claims data-based analysis of the influence of individual and regional characteristics on the utilisation of long-term care by people with dementia in Baden-Wurttemberg, Germany

**DOI:** 10.1186/s12877-019-1370-1

**Published:** 2019-12-19

**Authors:** Johanna Forstner, Michel Wensing, Jan Koetsenruijter, Pamela Wronski

**Affiliations:** Department for General Practice and Health Services Research, University Hospital Heidelberg, Im Neuenheimer Feld 130.3, Marsilius Arkaden, Turm West, 69120 Heidelberg, Germany

**Keywords:** Dementia, Long-term care, Informal care, Respite care, Regional variation, Baden-Wurttemberg, Multilevel analysis, Claims data

## Abstract

**Background:**

Challenges of future dementia care include increasing shortage of qualified healthcare providers and decreasing potential of informal care by relatives. In order to meet those challenges, changes in dementia care are needed. These changes should be based on data of both care utilisation and care supply. The aim of this study was to provide insight into individual and regional characteristics that influence the utilisation of long-term care by people with dementia.

**Methods:**

The study was a retrospective cross-sectional analysis of claims-based data and other available data referring to one index year. All data were aggregated for small geographic districts. The study population comprised people with a dementia diagnosis, 65 years and older in Baden-Wuerttemberg and insured by the largest health insurer. Utilisation of nursing home care, informal care, and respite care was analysed using binary coded logistic multilevel analyses.

**Results:**

Seventy nine thousand three hundred forty-nine people with dementia were included in the analyses. Nursing home care was used by 20.4%, informal care by 30.6%, and respite care by 3.5% of people with dementia. Individual characteristics that influence care utilisation included age, sex and the level of care dependency. The utilisation of informal care (OR = 1.713) and respite care (OR = 2.036) was higher in rural districts than in city districts. Respite care supply had an effect on the utilisation of respite care (OR = 1.173).

**Conclusions:**

The study found differences between districts in the utilisation of long-term care for dementia. These differences were largely explained by the composition of the population within the districts. An exception was the utilisation of respite care, which was higher in districts which have higher supply. Individual characteristics that influenced care utilisation are age, sex, level of care dependency and, with regard to informal care, comorbidity. Further research should be conducted on a small-area level, include further individual characteristics as well as other care and living forms.

## Background

In 2016, approx. 1.63 million people with dementia aged 65 and older were living in Germany [1]. About 50–70% of those affected suffer from Alzheimer’s disease, for which age is the strongest risk factor. In the group of people aged 65 and older, the prevalence of Alzheimer’s disease is 7.0%, with a duplication of prevalence rates in five-year increments for the age groups to 90 years old [2, 3]. Against the backdrop of demographic change, estimates predict an increase in the number of people with dementia to over two million by the year 2050 [2–5].

### Long-term care policies in Germany

To face these challenges, long-term care (LTC) in Germany is financed by social and mandatory private LTC insurance. Social LTC insurance covers most parts of the population (approx. 90%). In order to claim LTC benefits, insurants have to file an application to their insurance company. Then, an independent organisation assesses insurants’ eligibility for LTC benefits based on legally defined criteria focussing on physical need of help with activities and instrumental activities of daily living for at least 6 months [6–8]. It was only in 2017 that cognitive impairment has been added to these criteria [9].

Based on the amount of help needed, insurants are classified into a level of care dependency (LoCD). The LoCD determines the amount of benefits insurants can claim. LTC insurance is designed as part insurance cover, out-of-pocket payments are intended [7, 8].

The benefit package includes the following: home care in-kind benefits (for the remainder of this article referred to as ‘outpatient care’), home care in-cash benefits (hereafter referred to as ‘informal care’), inpatient care, day and night care (hereafter referred to as ‘respite care’, see Table 1), low-threshold support services, and professional caregivers for when an informal caregiver is on vacation [7].

**Table 1:** German policies and definitions of terms specific to German long-term care

Outpatient care	Outpatient care includes nursing, care and support services, and support with housekeeping
Informal care	Informal care includes in-cash benefits to compensate informal caregivers.
Respite care	Respite care is a term to describe any kind of temporary provision of care in order to give the caregiver temporary relief from caregiving [10]. In this article and based on German LTC insurance understanding, we define respite care as adult day care and adult night care, provided in a dedicated institution.
Levels of care dependency for people with dementia in 2013	In the year 2013, there were three LoCD and an additional LoCD 0 for people with a limited capability to manage their everyday life. With a higher LoCD the claim for benefits increases. A LoCD 0 does not allow claiming all kinds of LTC services. In the year 2013, amongst others, the following services were available in the different LoCD: LoCD 0: in-kind services in-cash services LoCD 1–3: in-kind services, in-cash services, nursing home care, respite care Hardship case: Hardship cases are people in LoCD 3 who receive more benefits due to severe need for LTC. [3, 6, 7, 11]
Long-Term Care Benefits Amendment Act	Entry into force: January 2002 Changes for people with dementia: first time possibility to claim financial aids through LTC insurance by people with dementia to use for care and support services; introduction of the so-called ‘LoCD 0’ [12]
Long-Term Care Further Development Act	Entry into force: July 2008 Changes for people with dementia: increase in the amount of benefits to claim [13]
Act to Realign Long-Term Care	Entry into force: January 2013 Changes for people with dementia: first time possibility to claim in-kind or in-cash benefits in home care for people with dementia in a LoCD 0 [14]
1st Act to Strengthen Long-Term Care	Entry into force: January 2015 Changes for people with dementia: first time possibility to claim respite care benefits, short-term care, and substitutional care (for when the informal caregiver is on vacation) [15]
2nd Act to Strengthen Long-Term Care	Entry into force: January 2017 Introduction of the new definition of the need of long-term care, considering not only physical but also cognitive impairments [9]

Table 1 provides an overview on German LTC terms and reforms.

### Future challenges in dementia care

A major challenge in dementia care is the question of how to provide optimal care for the increasing number of people with dementia. A large part of people with dementia choose to live in their own home and claim home care benefits, which is in line with the government aim “outpatient before inpatient” [16]. However, the potential of informal care is decreasing due to changing family- and acquisition structures [3]. Increasing severity of dementia often comes along with symptoms such as aggressiveness or wandering, exceeding the amount of support that informal caregivers are able to provide. Therefore, people with severe dementia tend to utilise professional care, often in institutional settings [3, 17–19] However, Germany is facing an increasing shortage of qualified personnel in nursing [20].

These challenges are especially affecting rural areas, as the elderly population tends to remain in rural areas whereas younger people move to urbanized areas. There are several studies that address regional differences in LTC supply and utilisation in Germany [3, 21–23] and internationally, especially in territorial states such as Canada, the United States or Australia [24–27]. For example, Mitchell et al. found that older adults in urban areas were more likely to use home care services [27]. Rudel et al. found that older adults living in rural areas perceive a lack of LTC supply [28]. Rothgang et al. found that the supply of nursing home beds and the number of staff in nursing homes and outpatient care services differs between districts in Germany. Furthermore, there are differences in the utilisation and capacity of nursing homes and outpatient care services, respectively [21]. There is evidence that care supply affects LTC service utilisation. Pilny and Stroka found that the supply of nursing home beds is linked to an increasing utilisation of nursing home care whereas the utilisation of informal care decreases [29]. Rothgang et al. showed that both inpatient and outpatient care supply affect utilisation, respectively. Furthermore, they identified unemployment as a factor that affects the utilisation of informal care [21].

Therefore, we expected that there are regional differences in the utilisation of LTC services by people with dementia that depend on not only individual characteristics, but also on regional factors. However, in Germany little is known on the influence of individual and regional factors on care utilisation. The aim of this study therefore is to provide insight into individual and regional characteristics that influence the utilisation of LTC by people with dementia.

## Methods

### Study design

The study presented here is embedded in a larger project which was initiated by the Ministry of Social Affairs and Integration of Baden-Wurttemberg, a federal state in southern Germany, and which aims at developing a concept for cross-sectoral care in three districts in Baden-Wurttemberg, based on elaborate analyses of the current and prospective state of care [30]. The study presented here is a retrospective cross-sectional analysis of the year 2013 using claims-based data, data of the Long-Term Care Statistics, and other secondary data. Claims data are extensive and available on a small-area level, which is difficult to achieve with primary data.

### Study population

The study population comprises people with a dementia diagnosis, insured with the health insurance company “Allgemeine Ortskrankenkasse” (AOK) Baden-Wurttemberg, aged 65 and older, who utilised any statutory health insurance (SHI) service in 2013, and whose place of residence is in Baden-Wurttemberg. The AOK insured approx. 43% of all SHI-insured persons in Baden-Wurttemberg in 2013 [31].

**Fig. 1:**
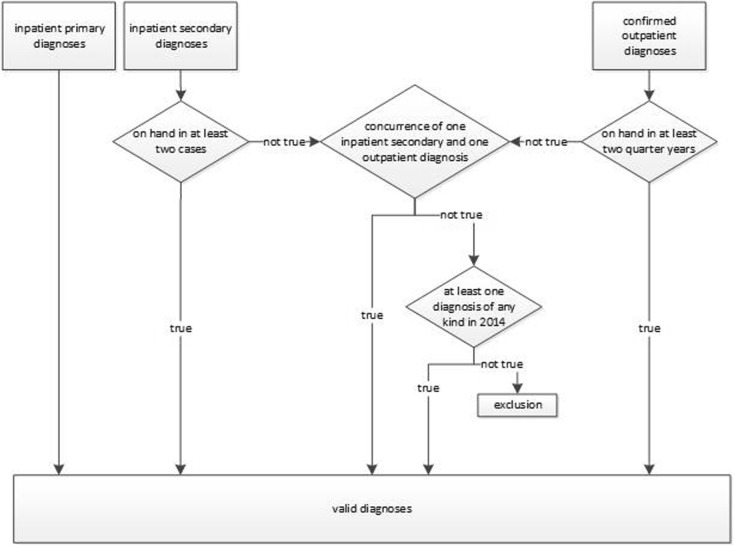
Internal validation of dementia diagnoses

Based on the literature, we included individuals with diagnosis of dementia, which was defined using the International Classification of Diseases (ICD) Codes F00, F01, F02, F03, F05.1, G30, G31.0 and G31.82 [3, 32–34]. Several measures were taken in order to enhance the validity of diagnoses. All hospital discharge primary diagnoses were considered valid [35]. Hospital discharge secondary diagnoses and confirmed outpatient diagnoses were only included in the analyses if one of the following criteria was met: documentation in at least two quarters for outpatient diagnoses or cases for hospital discharge secondary diagnoses or a combination of at least one outpatient and at least one hospital discharge secondary diagnosis. If only one diagnosis was documented in 2013, people were included in the analyses if at least one diagnosis was documented in 2014 in order to include incident people in the analyses. The intersecting set of diagnoses that meet all criteria described above constitutes our study population (see Fig. 1).

### Data sources

Patient data, diagnoses and LTC data were extracted from claims-based data from AOK Baden-Wurttemberg for the years 2013 (observation period) and 2014 (validation period). The use of the data set for analyses of the evaluation of general practitioners-centred care [36] for our analysis was approved by the AOK Baden-Wurttemberg. Data provision was carried out according to European data protection laws. Other aggregated health data and regional administrative on a district level were collected from the INKAR database (Indicators and Maps for Spatial and Urban Development) of the Federal Institute for Building, Urban Affairs and Spatial Research, the Health Atlas BW of the Baden-Wurttemberg State Health Office, the Regional Database Germany GENESIS of the Federal Statistical Office and the statistical offices of the Lander, and the Long-Term Care Statistics Baden-Wurttemberg, which was retrieved from the Regional Database GENESIS and the Statistical Office of the Federal State Baden-Wurttemberg. Except of AOK-data, all data are publicly available and were retrieved online for the year 2013, Long-Term Care Statistics data were extracted for the due date 31st December 2013. On-site data retention, study-related data preparation and data linkage were carried out using MariaDB Server 10.1.5 (64 Bit). Claims data were linked to district-level data via a four-digit identification code for districts.

### Measures

The outcome in the analyses was the utilisation of several long-term care forms: nursing home, outpatient care, informal care and respite care (definition see Table 1). In order to reduce complexity in data analysis, the type of care was operationalised using various binary coded variables. Being in a nursing home is exclusive, no outpatient care can be provided. All other types of LTC are provided in the outpatient sector and are not exclusive. All LTC data was extracted for the month of December 2013 in order to match the cross-sectional data from the Long-Term Care Statistics. LTC provision for January to November 2013 therefore was not considered.

Nursing home utilisation was coded “1” for full nursing home care at the time of data extraction (December 2013). Patients who had full nursing home care and outpatient care at the same time were interpreted as short-term care patients and assigned to the outpatient care sector. Utilisation of all other care forms was approximated by costs.

The following independent variables on an individual level were included in the analyses: age, sex, level of care dependency (LoCD), and the Charlson Comorbidity Index (CCI). Independent variables on a district level included are: district type (a model to depict population density in districts and district free cities, the one used for these analyses is a variation of the original four-category model [37]: the categories for rural districts were combined into one category (“rural district”), as there are only few rural districts within Baden-Wurttemberg), urban/rural area, unemployment rate, average household income, and care supply of the different care forms, their capacity utilisation rate as well as the number of inpatient and outpatient staff.

For the analysis of respite care and nursing home, LoCD 1 was used as the reference category instead of LoCD 0, as in 2013 compensation for respite care and nursing home care was not possible for patients with a LoCD 0. This was only introduced in 2015 as part of a major LTC reform with the intention to expand the entitlement for claims within LTC-insurance for people with cognitive impairments (see Table 1) [11].

Age categories were constructed as 5-year age categories from age 65 to 95 and 95 and older in order to depict morbidity. Comorbidities are operationalised using the CCI subtracted by the factor 1 which is used for dementia when calculating the CCI. A categorisation of the CCI into mild, moderate and severe documented comorbidity was made according to Huang et al. [38, 39].

Supply of respite care is defined according to indicator 6.20 of the Health Monitoring of the Lander as care spots per 1000 inhabitants age 65 years and older. Supply of outpatient care is defined as the number of outpatient care services per 1000 inhabitants who are 65 years and older. Outpatient capacity utilisation rate is defined as the average number of people in need of LTC as defined by law (see Table 1) per outpatient care service; nursing home care/ respite care capacity utilisation rate is defined as the number of people in need of LTC in nursing home care/ respite care per number of care spots in nursing home care/ respite care. Variables for staff are defined as the average number of staff per outpatient care service and nursing home, respectively. All definitions for respite care concern day as well as night care if not stated otherwise. All variables on care supply, capacity utilisation rate and staff are based on the Long-Term Care Statistics and are calculated on the basis of all people in need of LTC and not only people with dementia.

### Data analysis

Descriptive analyses of the study population and the districts of Baden-Wuerttemberg were conducted. In order to consider the hierarchical structure with individuals nested in districts, multiple binary logistic multilevel regression analyses were conducted for each type of utilisation of the three long-term care forms. Persons who did not utilise any LTC services were set as the reference group for all analyses. Independent variables were included as fixed effects and the subject district was set as random intercept.

An alpha-level of *p* < 0.05 was used to test for significance. However, as all analyses are explorative, the *p*-value should be seen as descriptive. Statistical analyses were conducted using IBM SPSS Version 24. A reporting standard for secondary data was used to assure transparency of analyses [40].

## Results

### Study population

The study population comprised 79,349 individuals. Two thirds of the study population were female (67.1%). The mean age was 82.8 years (standard deviation (SD) 7.06) and the oldest included person was 107 years. 63.1% were in need of LTC and therefore classified in a LoCD, and 51.6% claimed any of the LTC services included. 36.9% of the study population were not in need of LTC. Most included individuals lived in an urban area. Table 2 gives an overview on the results of the descriptive analysis of individuals.

**Table 2:** Characteristics of the study population

	Individuals (*n* = 79,349)
Age^a^	82.8 (7.06); 65–107)
Age in categories [n(%)]	
65–69 years	2820 (3.6)
70–74 years	7500 (9.5)
75–79 years	15,294 (19.3)
80–84 years	20,161 (25.4)
85–89 years	19,289 (24.3)
90–94 years	11,571 (14.6)
95 years and older	2714 (3.4)
Sex [n(%)]	
Male	26,115 (32.9)
Female	53,234 (67.1)
LoCD [n(%)]	
No LoCD	29,263 (36.9)
LoCD 0	2444 (3.1)
LoCD 1	18,604 (23.4)
LoCD 2	19,319 (24.3)
LoCD 3	9609 (12.1)
Hardship case	110 (0.1)
Comorbidity (CCI) [n(%)]	
none	19,710 (24.8)
mild	24,783 (31.2)
moderate	18,055 (22.8)
severe	16,801 (21.2)
Area [n(%)]	
City district	11,467 (14.5)
Urban district	54,942 (69.2)
Rural district	12,940 (16.3)
No LTC services [n(%)]	38,388 (48.4)
Nursing home care [n(%)]	16,159 (20.4)
Outpatient care [n(%)]	852 (1.1)
Informal care [n(%)]	24,281 (30.6)
Respite care [n(%)]	2812 (3.5)

^a^[mean (standard deviation (SD)); range]

The administrative prevalence of dementia in people insured with the AOK Baden-Wuerttemberg and aged 65 and older was 9.08%. The prevalence in females (10.2%) was higher than the prevalence in men (7.4%). The median for documented comorbidities lied at a CCI of 2, which means that half of the study population had no or little documented comorbidities. The most common documented comorbidities included hypertension, disorders of lipoprotein metabolism, Diabetes mellitus type 2, urinary incontinence, depressive episode, heart failure, chronic ischemic heart disease, back pain, gonarthrosis, and accommodation and refraction errors.

From the districts included, 8 were district-free cities, 27 are urban districts and 9 were rural districts. Table 3 shows the results of the descriptive analysis of districts.

**Table 3:** Characteristics of districts

	Districts (*N* = 44)
Area [n (%)]	
City district	8 (18.2)
Urban district	27 (61.4)
Rural district	9 (20.5)
Unemployment rate ^a^	4.1 (1.1); 2.8–8
Employment rate ^a^	81.9 (5); 59.7–87.1
Mean household income ^a^	1889.3 (271.6); 1633.7–3466.9
Full nursing home spots available ^ab^	49.1 (9.1); 35–69.8
Respite care spots available ^ab^	3 (1.2); 1–6.5
Respite day care spots available ^ab^	3 (1.2); 1–6.1
Number of outpatient care services ^ab^	0.6 (0.1); 0.3–0.9)
Outpatient capacity utilisation rate ^ab^	56.7 (12.6); 32.6–90.7
Nursing home capacity utilisation rate^ab^	88.8 (3.4); 80.6–95.9
Respite care capacity utilisation rate ^ab^	133.3 (31.5); 72.6–216.3
Inpatient staff ^ab^	55.6 (10.6); 38.5–85.4
Outpatient staff ^ab^	28.4 (5.8); 18.9–45.6

^a^[mean (SD); range]

^b^per 1000 inhabitants aged 65 years and older

### The influence of individual and regional characteristics on the utilisation of long-term care services

About one fifth (20.4%) of the study population lived in a nursing home, 30.6% were being taken care of by informal caregivers, 1.1% received care services by an outpatient care service and 3.5% utilised respite care. Almost half of the study population (48.4%) did not utilise any of the LTC services mentioned above (Table 2).

In the analysis of LTC utilisation, the null models revealed an intra class correlation (ICC) of 0.062 for the utilisation of nursing home care, an ICC of 0.020 for the utilisation of informal care and an ICC of 0.112 for the utilisation of respite care. This shows the necessity of computing multilevel analyses in order to identify those regional characteristics that cause variance. Therefore, multilevel models were constructed for the outcomes nursing home, informal care, and respite care (see Table 4). No model was computed for the utilisation of outpatient care services as the comparatively low number of cases (*n* = 852) indicates that it does not play a major role in dementia care. Unemployment rate, outpatient capacity utilisation rate, nursing home capacity utilisation rate, outpatient staff, and inpatient staff did not have a significant effect on care utilisation and therefore were excluded from the final models.

**Table 4:** Multilevel analysis of the utilisation of nursing home care, informal care and respite care

	Nursing home			Informal care			Respite care		
	OR^a^	p^a^	95% CI^a^	OR	p	95% CI	OR	p	95% CI
Age	1.005	0.02	[1.001;1.009]	0.984	0.00	[0.981;0.988]	0.962	0.00	[0.956;0.969]
Sex (reference: male)	1.404	0.00	[1.315;1.499]	0.850	0.00	[0.804;0.899]	1.080	0.12	[0.978;1.193]
LoCD (reference: LoCD 0/ LoCD 1^b^)									
LoCD 1	–	–	–	0.384	0.00	[0.339;0.436]	–	–	–
LoCD 2	2.601	0.00	[2.442;2.770]	0.368	0.00	[0.324;0.418]	1.622	0.00	[1.475;1.782]
LoCD 3	3.607	0.00	[3.345;3.889]	0.307	0.00	[0.268;0.352]	0.807	0.00	[0.702;0.929]
Hardship case	5.723	0.00	[3.359;9.751]	0.144	0.00	[0.072;0.287]	0.206	0.12	[0.207;1.569]
CCI categories (reference: no comorbidity)									
Mild	1.049	0.20	[0.975;1.129]	1.058	0.09	[0.990;1.130]	1.081	0.18	[0.963;1.212]
Moderate	1.061	0.14	[0.979;1.148]	1.134	0.00	[1.056;1.218]	0.945	0.38	[0.831;1.074]
Severe	1.104	0.01	[1.017;1.200]	1.261	0.00	[1.171;1.357]	0.880	0.06	[0.770;1.005]
Area (reference: City district)									
Urban districts	1.335	0.24	[0.817;2.181]	1.360	0.05	[0.992;1.864]	1.566	0.00	[1.116;2.196]
Rural districts	1.515	0.12	[0.892;2.573]	1.713	0.00	[1.220;2.406]	2.036	0.00	[1.417;2.926]
Mean household income	1.000	0.71	[1.000;1.001]	1.000	0.86	[1.000;1.000]	1.000	0.45	[1.000;1.001]
Nursing home care supply	1.003	0.76	[0.982;1.025]	0.992	0.27	[0.979;1.006]	0.986	0.06	[0.972;1.001]
Respite care supply	0.924	0.25	[0.808; 1.057]	0.954	0.28	[0.876;1.040]	1.173	0.00	[1.075;1.280]
Respite care capacity utilisation rate	0.995	0.06	[0.990;1.000]	0.997	0.09	[0.994;1.001]	0.998	0.19	[0.994;1.001]
ICC^a^	0.062	0.00		0.020	0.00		0.112	0.00	
n	54,547			62,699			41,200		

^a^*ICC* Intra Class Correlation, *OR* Odds Ratio, *p* Alpha-Level 0.05, *CI* confidence interval

^b^for nursing home care and respite care the LoCD 1 was chosen as a reference, as people with dementia were not eligible for those care types in the LoCD 0 in 2013

The analyses for the outcome nursing home (*n* = 16,159) showed significant effects for almost all independent variables on an individual level. Women were 1.4 times more likely to live in a nursing home than men. Furthermore, the likelihood to live in a nursing home was higher with increasing age (odds ratio (OR) =1.005 per year), a high LoCD (for example LoCD 3 OR = 3.607 as compared to LoCD 1) and severe comorbidity (OR = 1.104 as compared to no comorbidity). Other categories of the CCI were not significantly different from having no comorbidities. When controlling for variables on an individual level, no significant effect was found for variables on a district level.

All variables on an individual level showed a significant effect on informal care. The likelihood to be taken care of by informal caregivers decreased with higher age (OR = 0.984), a high LoCD (OR = 0.307 for LoCD 3 as compared to LoCD 0), and was higher in men than in women (OR = 0.850). However, the likelihood increased with severe comorbidity (OR = 1.261 for severe comorbidity as compared to no comorbidity). Additionally, people living in rural districts were more likely to utilise informal care than people living in city districts (OR = 1.713).

The analysis of respite care shows a decreasing likelihood of utilisation with increasing age (OR = 0.962). When considering levels of care, the likelihood of utilisation was higher in LoCD 2 compared to LoCD 1 by the factor 1.622. In LoCD 3 compared to LoCD 1 however, the likelihood decreased (OR = 0.807). In addition, people with dementia with severe comorbidity were less likely to utilise respite care than those with no comorbidity (OR = 0.880). No significant effect was found for sex and other categories within the levels of care and comorbidity. However, several district level factors had a significant effect on the utilisation of respite care. The likelihood for utilisation was higher in both urban and rural areas than in city districts, respectively (OR = 1.566 for urban districts and OR = 2.036 in rural districts). Furthermore, an effect of the care supply on utilisation was found: an increasing supply came along with higher utilisation (OR = 1.173).

## Discussion

The intracluster correlations demonstrated differences between regions in the utilisation of LTC. These differences were largely explained by the composition of the population within the regions. An exception was the utilisation of respite care, which was higher in regions with higher supply of such care.

The prevalence rate of dementia for people aged 65 and older was 9.08% in our study and thereby approximately two percentage points higher than older studies on dementia prevalence [3, 41, 42]. This may be explained by the fact that the meta-analyses by Bickel and Prince et al. included studies mostly conducted before the year 2000. For instance, the prevalence rates by Doblhammer et al. were based on data from the year 2007.

The increasing utilisation of formal care with higher age reflects the results from previous studies [18, 43]. Furthermore, our results show lower informal care utilisation with higher age. Other studies indicate an increase in self-reported informal care time with higher age [43]. In our population, informal care was measured based on claims-data instead of care time. As the option for financial support of informal care was only introduced in 2013, the rather younger people with dementia used this option first rather than older people with a possibly longer course of disease who tend to utilise more formal care forms. Also, women tend to be cared for in formal care. This could be explained by studies that show that women are often older and without a spouse [3, 44].

Our results show that approximately two thirds of the study population were eligible to claim LTC insurance benefits, but only half of the study population utilised any of the LTC services included in the analyses. Schulze et al. [45] present results showing that approx. 70% of people with early stages of dementia were not in need of LTC. Schwarzkopf and colleagues show that increasing severity of dementia leads to an increasing utilisation of formal care services [46]. People with dementia with a low LoCD are possibly people with mild dementia and are in need of other support services rather than those LTC services considered in this study.

Concerning regional factors influencing LTC utilisation, both Pilny and Stroka [29] and Rothgang et al. [21] found an effect of the inpatient and outpatient care supply on care utilisation. However, in our study only utilisation of respite care was affected by care supply. In the other studies, different definitions for the variables were used and the study population did not focus on people with dementia. Furthermore, respite care for people with dementia in a LoCD 0 was only introduced in 2013. Supply of respite care has been relatively low in Germany; its expansion has been pushed by the government, as supply was considered to be too low compared to its demand [11].

An expected benefit of respite care is delay of nursing home placement, which has been subject of many studies. Weyerer et al. [47] and Gaugler et al. [48] suggest that respite care can delay institutionalization. In a systematic review Fields et al. however found such no effect [49]. Nevertheless, respite care reduces informal caregiver burden [49, 50]. The German government took this into consideration this by opening the utilisation of respite care to people with dementia in a LoCD 0 with the major LTC reform in 2015.

Some findings differed from previous studies. For instance, Rothgang et al. suggested unemployment rate as an indicator for time resources available for informal care [21]. We therefore tested whether the unemployment rate had an effect on the utilisation of informal care but no effect was found. Furthermore, our results indicate an effect of rurality on the utilisation of informal and respite care. This differs from the findings of Rothgang et al. [21] and Donath et al. [51]. However, when comparing the results, one should consider that Rothgang et al. investigated people in need of LTC in general and not only people with dementia. Contrary to the results of Unger et al. [52] no effect of income on the utilisation of different care forms was found. However, this must be interpreted with caution as data on income in this study were only available on a regional level. Transferring this effect on individuals could lead to ecological fallacy.

### Limitations

This paper provided insight into the influences of individual and regional characteristics on the utilisation of different LTC forms. There are some limitations to consider.

First, the care forms examined in the study do not cover the full spectrum of care options for people with dementia. For instance, care services such as voluntary care were not covered [23]. Inclusion of further living and care forms in the analyses was not possible due to data availability within the Long-Term Care Statistics.

Second, claims-based data are not collected for academic purposes. Hence, data on an individual level such as socioeconomic status, income or family status are not recorded and validity of dementia diagnoses is limited due to lacking clinical data. Furthermore, claims-based data and ICD-codes do not allow differentiation between different stages of dementia severity. These limitations have already been discussed sufficiently in the literature [28, 32, 53]. However, the study design using secondary data reduced the risk of selection bias and enabled us to investigate a large study population that included groups that are difficult to access and which could not be achieved using primary collected data. Furthermore, recall bias does not apply to study designs based on administrative data [53].

Third, the operationalisation of ‘region’ is based on administrative districts which do not necessarily reflect the everyday reality of how health care supply is utilised as in the German health care system care utilisation takes place across regions.

Fourth, as only 44 districts were included in the analysis, only few variables on a district level could be included considering the degrees of freedom.

Lastly, external validity of the results is limited as we only used data from one insurer. The AOK historically insured people from a lower socioeconomic background and its community of insured people can differ from those of other statutory or private health insurance companies [54, 55]. However, we were able to use a full sample of people with dementia within the AOK which is the biggest health insurer in Germany’s third-largest province.

## Conclusion

The analyses in this paper give an overview on LTC of people with dementia in Germany using the example of the federal state Baden-Wurttemberg. Our data suggest that a further expansion of respite care might be necessary, however more recent data on supply and utilisation should be considered.

Secondary data analyses can help to get an initial overview on the state of care of people with dementia. However, in order to map the effects on LTC in a differentiated and realistic manner, further personal characteristics and other care and living forms, such as outpatient flat-sharing communities should be included. Then, differences at a small-area level should be examined and linked with primary data. Ulrich et al. [56] propose to include appraisal of local stakeholders in health care in order to interpret results against the background of local care structures. In order to increase external validity, analyses using data from more than one health insurance company could be considered. Appropriate indicators should be developed to detect unintentional variation in LTC of people with dementia.

## Data Availability

All data other than claims-based data are publicly available as described in the methods section. AOK data is only available at the data owner on inquiry.

## Abbreviations

AOK: Allgemeine Ortskrankenkasse, German health insurance company
CCI: Charlson comorbidity index
ICC: Intra-Class-Correlation
ICD: International Classification of Diseases
INKAR: Indicators and Maps for Spatial and Urban Development *(Indikatoren und Karten zur Stadt- und Raumentwicklung)*
LocD: Level of care dependency (*Pflegestufe*)
LTC: Long-term care
OR: Odds ratio
SD: Standard deviation
SHI: Statutory health insurance
